# Comparing types and patterns: A context-oriented approach to densification in Switzerland and the Netherlands

**DOI:** 10.1177/23998083221142198

**Published:** 2022-12-02

**Authors:** Vera Götze, Mathias Jehling

**Affiliations:** Institute of Geography, 27210 University of Bern, Switzerland; Leibniz Institute of Ecological Urban and Regional Development (IOER), Germany

**Keywords:** urban densification, land policy evaluation, cluster analysis, relative urban metrics

## Abstract

While governments worldwide develop policies to promote urban densification, critics point to possible negative effects of densification on social sustainability. The occurrence and distribution of these negative social effects are strongly influenced by land policies. This makes it crucial to monitor the role of land policies and understand what processes shape urban development in the context of densification. To do so, detailed, large-scale international comparisons of densification patterns, including building and social changes, are needed. We address this issue by introducing a method to measure and compare urban development in two countries with contrasting planning systems: the Netherlands, where public actors play a strong and active role, and Switzerland, where strong private property titles and a highly democratic planning system are prevailing. Our GIS-based method analyses densification processes within their surrounding morphological and socio-demographic context. A k-proto cluster analysis on highly detailed spatial and statistical data based on housing units, covering 2011–2019, results in five densification types. The distribution of these types reveals different patterns in the two city regions of Utrecht (NL) and Bern (CH). Most strikingly, contiguous redevelopments frequently occurred in Utrecht but hardly in Bern, pointing at possible advantages for Dutch municipalities to intervene in property rights. While having developed an empirical basis in this study, future research that refines the analysis of the legal, planning and ownership conditions underlying the identified densification patterns can contribute significantly to policy evaluation.

## Introduction

While governments worldwide rely on densification policies to reduce land consumption from urban growth, possible ecological, economic and social effects of urban densification are being widely discussed (Berghauser Pont et al., 2021). Many of these effects are not inherent to densification – they manifest in some cases but not in others. For instance, some types of densification are associated with the decrease of green spaces, while others seem to increase their amount (Wellmann et al., 2020). Since densification types come with different ecological, economic and social effects, it is important to understand under which conditions these types occur.

Different forms of densification can be observed both between and within urban regions (Jehling et al., 2020; Angel et al., 2021). However, studies on the effects of densification seldomly differentiate its location within a region or distinguish varying transformation processes (Bibby et al.,2020; Jehling et al., 2020). Often, these studies are too coarse to show intra-regional differences, thereby hiding the local factors that shape urban development (Bibby et al., 2020; Broitman and Koomen, 2020). Revealing these influencing factors is crucial to understanding under which conditions governments can use land policies to steer densification in a more desired direction (Nabielek, 2011; Jehling et al., 2020).

Land policies strongly influence densification and thus the occurrence and distribution of its effects. By determining which planning instruments municipalities have at their disposal (Gerber et al., 2018), they change power relationships between public and private actors, ultimately reflected in the evolving patterns of urban densification (Charmes and Keil, 2015). More concretely, land policies determine the extent to which urban development takes place on private or public land, whether fragmented landownership impedes brownfield transformations, but also what forms of housing are constructed and for whom. As governments pursue varying land policies, different densification patterns evolve.

Research on the effect of the institutional context (of which land policies are part) on urban densification is generally limited to small-scale, qualitative case studies. Single-case studies uncover, for instance, how increases in rent after redevelopment are anchored in Swiss institutions (Debrunner et al., 2020) or how, in the Netherlands, high densities in redevelopment projects are related to land acquisition costs (Buitelaar and Segeren, 2011). In addition, comparative case studies have helped explain causal relationships between national policies and urban development patterns (Tennekes et al., 2015; Giddings and Rogerson, 2021). Few studies apply this knowledge to explain densification patterns on a larger scale (Clessens et al., 2020; Jehling et al., 2020). However, they often focus on one region only. As an exception, Jehling and Hecht (Jehling and Hecht, 2021) compare urban morphology in Germany and France – though not regarding urban densification or social changes.

In light of the above, there is a need for analyses that differentiate between various forms of densification – including building and social changes – within metropolitan regions to nuance the debate on social effects. Comparisons of these analyses further shed light on the conditions under which densification evolves, and the role of institutional contexts in steering it. Thus the aims of this paper are to (1) present and test a comparative method to analyse densification as a process within existing social and built urban contexts and (2) develop hypotheses for the role of institutional contexts in shaping densification. These aims can be operationalised with the following questions: On the scale of new housing units and their urban context, ‘What types of densification emerge in different institutional contexts?’; regarding the distribution of these types, ‘Which patterns of densification become visible within city regions and across national institutional contexts?’. To answer these questions, we analyse densification in two city regions across two countries with contrasting institutional arrangements – Utrecht in the Netherlands, where public actors play a strong and active role, and Bern in Switzerland, where strong private property titles and a highly democratic planning system are prevailing. The approach is based on a particular interest in how urban contexts are affected by densification. Densification is thus measured in terms of (1) how new housing deviates from existing contexts regarding morphology, (2) how residents of newly constructed housing deviate from existing residents regarding socio-demographic variables, and (3) procedural metrics regarding the average project size in square meters and the process through which the new housing units emerged, that is, transformation of brownfields or urban green spaces, or (soft) densification in existing residential areas (Claassens et al., 2020). Using a k-proto cluster analysis (Huang, 1998), we identify and define five densification types and compare their occurrence between, as well as within the two regions. Observed differences in these patterns allow us to build hypotheses on the institutional conditions that shape urban development in the context of densification in the two case regions.

## Institutions and urban development

Urban development, understood as a result of human actions, happens within a web of existing institutions (Gerber et al., 2018). Institutions are formal rules and informal practices or narratives impacting actor agency (Lowndes and Roberts, 2013). While informal institutions represent implicit norms, such as planning cultures (Buitelaar and Bregman, 2016). Formal institutions legally constrain actors through procedural rules pertaining to planning, property rights, the distribution of competencies between government agencies, and land policies (Gerber et al., 2018, p. 13). We define land policy as the strategic application of political and legal measures by a municipality to deal with the problem of land scarcity (Hartmann and Spit, 2015). This definition introduces governments as political actors that pursue goals, making densification ‘a deliberate strategy, an unintended consequence of planning policy or the lack of enforcement’ (Dembski et al., 2020, p. 211)*.* Put differently, the interplay between institutions and interests affects urban development and its correspondent benefits and drawbacks. Together, institutions and interests determine ‘[w]ho owns the land, who decides which land uses should prevail, who appropriates the benefits of land uses, who suffers the burden […]’ (Davy, 2012, p. 68). Consequently, this informs our understanding of densification, its spatial distribution, transformation processes, and resulting housing forms.

This sets the institutional context as a known cause of which we want to investigate the spatial effect (Rohlfing, 2012). To do so, we study cases with a high variation on the cause, as this ‘permits the identification of potential independent variables that would be made subject to a subsequent hypothesis test’ (Rohlfing, 2012, p. 65). This implies choosing countries with contrasting institutional contexts and developing working hypotheses that explain observed differences in densification.

## Methods, case selection and data

### Methods

Our methodological approach consists of two steps; first we assign densification metrics to individual housing units based on harmonised data, and subsequently we identify densification types. In the context of this study, a housing unit is an individual, self-contained residential unit and can represent an apartment or a single-family house.

#### Relative metrics for social and built densification

For each new housing unit within the cases, we distinguish whether it was created through expansion or densification (following Hecht et al., 2019). Expansion encompasses all residential addresses constructed after a specific date (t_0_) on non-urban land, for example, agricultural land, forests, water bodies and other natural areas. These developments are excluded. The remaining developments on existing urban land fall into the densification category (following Broitman and Koomen, 2015). For addresses on a plot labelled ‘construction site’ in t_0_, we proceed with land use information from previous years (t_-1_).

Within the densification category, we further distinguish transformation processes using land use information from t_0_, that is, ‘transformation of urban green spaces’, ‘transformation of brown- and greyfields’, ‘densification in residential areas’, and ‘soft densification’ (Claassens et al., 2020) (see Figure S1). ‘Soft densification’ includes the transformation of non-residential addresses (such as offices, shops or attics) into apartments, the subdivision of apartments, and the expansion of existing buildings (Bibby et al., 2020).

The social metrics cover age structure, household size, living space and population density. These are measured at the highest possible resolution and describe the state after densification happened (t_1_). The age distribution is measured per hectare and is expressed as shares of children, students and elderly (Jehling et al., 2018). Population density is also measured per hectare (following Jehling and Hecht, 2021). Furthermore, to measure possible effects of crowding (Burton, 2000), we include apartment sizes (relating to individual addresses) and living space per person, expressed as the sum of apartment sizes divided by the number of inhabitants per hectare. In addition to social metrics, morphological metrics include building densities, such as floor space index, ground space index, and average building heights, measured at street block resolution.

**Figure 1.**
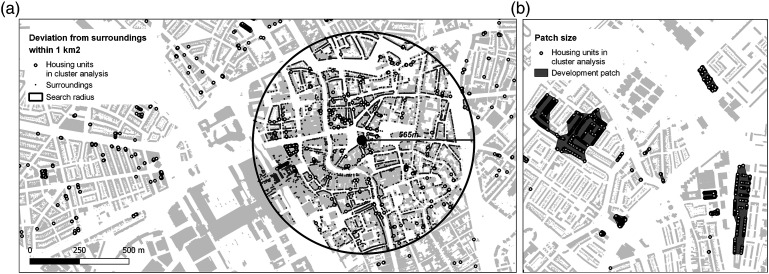
(A) Deviation from urban context: New housing units are analysed in relation to all pre-existing surrounding housing units (constructed before 2011) within a 1 km^2^ circle; (B) Patch size of densification projects (Utrecht city centre) (author’s work; data source: ©Kadaster).

The patch size of a densification project is measured by aggregating adjoining housing units into polygons (following Eggimann et al., 2020), using an empirically determined cut-off distance of 60 metres, that allows for the best possible separation of housing units in the case regions (Figure 1(b)). Separate units that are not part of a larger project are assigned a default patch size of 100 m^2^.

Finally, to take into account the social and built context, new housing units are described using relative metrics: Each new housing unit is characterised by its deviation from existing housing units within a 1 km^2^ circle (following Van Leeuwen and Venema, 2021) (Figure 1(a)). We express average differences in terms of standard deviations. The social, morphological and relative metrics, together with the respective patch size and transformation process, are assigned to the individual housing unit.

#### Classification and interpretation of densification types

Before performing cluster analysis, dimensionality reduction is necessary to enhance clustering results and ease cluster interpretation (Wilmink and Uytterschaut, 1984). To that end, variables resulting from the pre-processing steps described above are subjected to a multiple correlation analysis. In this step, highly correlated variables are iteratively pruned from the dataset until no two variables are highly correlated. This is done in preparation of the computationally expensive k-proto cluster analysis. To a certain extent, k-proto clustering resembles the better known k-means method that has proven viable in urban analytics, especially when working with large datasets (Berghauser Pont et al., 2019; Bobkova et al., 2021). Both algorithms partition data into *k* clusters, within which observations resemble each other as much as possible. However, unlike k-means, the k-proto algorithm calculates the distance between two variables expressed as the sum of their Euclidean distance and a simple distance measure for categorical variables, weighted by a factor *γ* (Huang, 1998). The resulting clusters are described using mean values for continuous variables and modes for categorical variables. This alternative clustering algorithm allows us to include the categorical variable ‘transformation process’ in the classification. We perform a combined cluster analysis on all new housing units that were created through densification across the two case regions. The resulting types are therefore common for both cases and allow for cross-case comparison, including the prevalence of types by region and their spatial distribution within the regions.

For cluster validation, we visually assess the resulting classification by way of principal component analysis.

### Case selection

The Netherlands and Switzerland have been pursuing compact city policies for decades and institutionalised urban densification as a primary goal in 2012 (Dutch Ladder for Sustainable Urbanisation) and 2014 (revision Swiss Spatial Planning Act). However, the implementation of these policies strongly differs in the degree to which municipalities can steer urban development projects, the role of property rights, and their legitimisation.

In the Netherlands, municipalities play an active role in urban development – either through land purchases or partnerships with private developers (Meijer and Jonkman, 2020).

Especially public-private partnerships are becoming more frequent following decentralisation and the withdrawal of national funding for public land acquisition (Claassens et al., 2020).

In Switzerland, public–private partnerships for urban development are uncommon. Also, other forms of active land policy, such as public land ownership combined with long-term land leases, have only recently been gaining momentum (Gerber, 2016). Compared to the Netherlands, citizens (through direct voting on re-zoning proposals) and private land owners (through strongly protected property rights) both have strong veto rights to block densification. Densification lies in the hands of private developers, with public actors facilitating (Debrunner et al., 2020). This difference in stakeholder power balance is also apparent in the rental housing sector: while individual private investors in Switzerland own 50% of the country’s rental housing (BFS, 2020), the Dutch rental housing sector is dominated by corporations (70%) and institutional investors (15%) (SCP, 2020).

To analyse densification, a scope is required that encompasses its variety within functional urban regions (Jehling et al., 2020). Since Switzerland is a confederation, with each canton applying policies differently, we confine the analysis to one canton. Accordingly, data collection in the Netherlands is limited to one province. Bern in Switzerland and Utrecht in the Netherlands form the two cases – two rapidly growing regions that do not exhibit the exceptional trends of the leading metropoles of Zürich and Amsterdam.

### Data collection, harmonisation and metrics

Data on housing units is available at unit resolution for both countries starting in 2011 (t_0_ in this study) (Table 1). Morphological data is aggregated per street block using national topographical datasets. Data on age distribution and household sizes is available at a 100 m resolution until 2019 (t_1_) for both countries. Data harmonisation requires particular attention to land use. Swiss Area Statistics on land use are available on a hectare grid only, where the value of each grid cell is determined by the land use covered by its lower left coordinate. We engineered the Dutch data to an identical representation. The resulting crude resolution land use patterns required redefining what constitutes a ‘residential area’. Functions usually found in residential areas such as retail, smaller roads and public buildings are hence included in the category ‘residential area’. Finally, a harmonised, comparative set with metrics to characterise morphology, inhabitant structure and transformation processes of urban redevelopments in Utrecht and Bern is summarised in Table 1 (see Table S1 for summary statistics). The metrics indicating deviation (d_), patch size and transformation type are assigned to individual housing units and passed to the cluster analysis.

**Table 1.** Metrics for characterising densification on level of housing units based on harmonised data set. Except for ‘transformation type’, all metrics refer to 2019. Transformation type is determined with land use data from 2000, 2010 and 2015 (NL) and 1997, 2009 and 2018 (CH).

	Name	Relevance	Description	Aggregation level	Source
Social	pop_dens	Crowdedness	Inhabitants per hectare	Hectare grid	A
	per_kids	Compatibility with families	Share of children (age 14 and below) per hectare	Hectare grid	A
	per_students	Compatibility with students	Share of students (age 15–24) per hectare	Hectare grid	A
	per_elderly	Compatibility with elderly	Share of elderly (age 65 and above) per hectare	Hectare grid	A
	hh_size	Compatibility with families	Average household size per hectare	Hectare grid	A
	m2person	Crowdedness	Sum of apartment sizes divided by population per hectare	Hectare grid	A/B
Built	Layers	Building height	Average number of storeys per street block	Street block	B
	Fsi	Building density	Ratio between floor area and block area per street block	Street block	B
	Gsi	Loss of open space	Ratio between building footprint and block area per street block	Street block	B
	Aptsize	Crowdedness	Apartment sizes of individual apartments	Housing unit	B
Social	d_popdens	Deviation from surroundings	Deviation in population density from surroundings	Hectare grid	A
	d_kids	Deviation from surroundings	Deviation in share of children from surroundings	Hectare grid	A
	d_students	Deviation from surroundings	Deviation in share of students from surroundings	Hectare grid	A
	d_elderly	Deviation from surroundings	Deviation in share of elderly from surroundings	Hectare grid	A
Social	d_hh_size	Deviation from surroundings	Deviation in household size from surroundings	Hectare grid	A
	d_m2person	Deviation from surroundings	Deviation in living space per person from surroundings	Hectare grid	A/B
Built	d_layers	Deviation from surroundings	Deviation in building height from surroundings	Street block	B
	d_fsi	Deviation from surroundings	Deviation in floor space index from surroundings	Street block	B
	d_gsi	Deviation from surroundings	Deviation in ground space index from surroundings	Street block	B
	d_aptsize	Deviation from surroundings	Deviation in apartment size from surroundings	Housing unit	B
Process	Patchsize	Transformation process	Size of densification project in m^2^	Housing unit	B
	transformation type	Transformation process	Factor with levels: transformation of brownfields/greyfields, transformation of urban green, densification on residential areas, soft densification	Hectare grid	C

A – STATPOP2019, BFS GEOSTAT Switzerland (Downloaded November 2, 2021); Statistische gegevens per vierkant 2020, CBS Netherlands (Downloaded September 23, 2021)

B – Bundesamt für Statistik; Eidg. Gebäude-und Wohnungsregister Switzerland (Downloaded February 8, 2021); Basisregistratie Adressen en Gebouwen (BAG) kadaster Netherlands (Downloaded July 27, 2021)

C – Arealstatistik, BFS GEOSTAT Switzerland (Downloaded March 4, 2021), Bestand Bodemgebruik, CBS Netherlands (Downloaded March 4, 2021)

## Results

**Figure 2.**
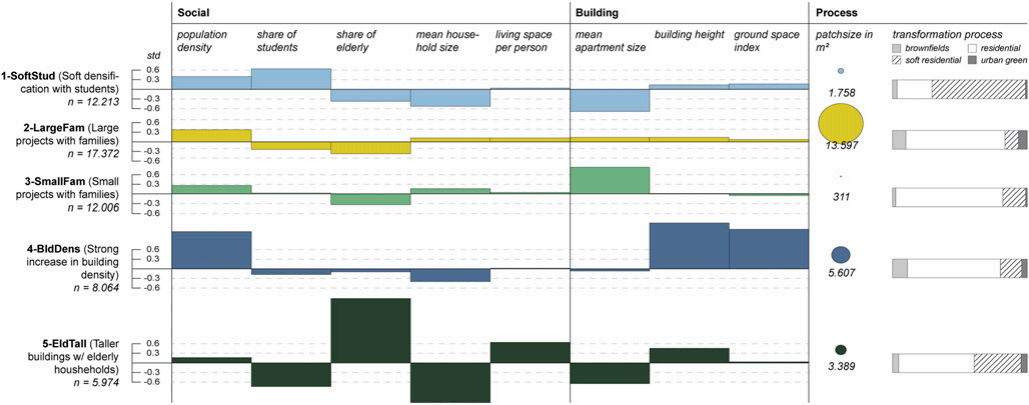
Densification types with the mean deviation of the associated housing units from their social and built surroundings and their process with average patch size and the distribution of transformation processes. We further differentiate the characterisation of densification types by comparing the variables’ distribution within these types (see Figure S4).

K-prototype clustering detects five densification types, which we describe in Figure 2. Broadly, the results show that densification yields higher population densities, smaller apartment sizes, smaller household sizes and smaller shares of elderly than the existing urban context (See Appendix with correlation analysis for dimensionality reduction (Figure S2) and Figure S3 for the choice of *k*).

The first cluster, *SoftStud*, contains primarily soft densification and conforms to the general observations, albeit with a higher share of students (std = 0.64).

The second cluster, *LargeFam*, describes large-scale densification projects. Though not differing strongly from its surroundings in terms of demography or morphology, it is one of two types where household and apartment sizes are on average larger than in their surroundings. *LargeFam* forms the largest of the five clusters, containing more than 30% of the housing units that entered the cluster analysis. Most transformations of brownfields and greyfields fall into this cluster.

In cluster three, *SmallFam*, household sizes are larger than their vicinity average, and it is the only cluster with substantially (std = 0.83) larger apartment sizes. Its makeup is mostly housing units within small-scale projects in existing residential areas.

Cluster four, *BldDens*, describes a densification type with unusually tall and densely built constructions (building height with std = 1.43; ground space index with std = 1.23), also featuring higher population densities (std = 1.16). Densification in residential areas and, to a lesser degree, transformations of brownfields fall into this type.

Cluster five, *EldTall*, describes a densification type with remarkably high shares of small, elderly households in much smaller apartments and buildings somewhat taller than their vicinity averages (share of elderly with std = 2.01; household size with std = −1.24). Here, population density increased only slightly as residents take up an unusually large amount of living space (std = 0.64). Just as in type one, this densification type is largely, but not predominantly, a result of soft densification.

### Prevalence of densification types per region

**Figure 3.**
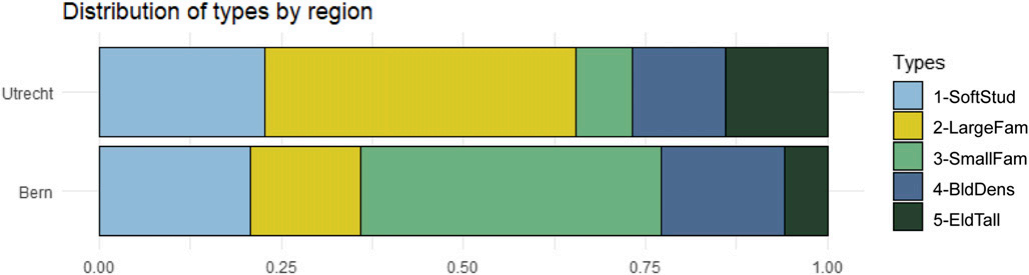
Distribution of types in Bern and Utrecht.

In this study, we cover a total of 55.000 housing units constructed on urban land between 2011 and 2019 (32.000 in Utrecht and 23.000 in Bern). These are fractions of the total net increases in the number of housing units in the same period (63.000 in Utrecht and 50.000 in Bern). The densification types are distributed differently in the two regions (see Figure 3).

Most of the housing units in *2-LargeFam* and *5-EldTall* were constructed in Utrecht, where they make up for around 60% of new housing units. In Bern, the most prevalent type is *3-SmallFam*, accounting for circa 40% of the urban development in our dataset. This densification type is typical for Bern; it hardly occurs in Utrecht.

### Spatial distribution of densification types by case

**Figure 4.**
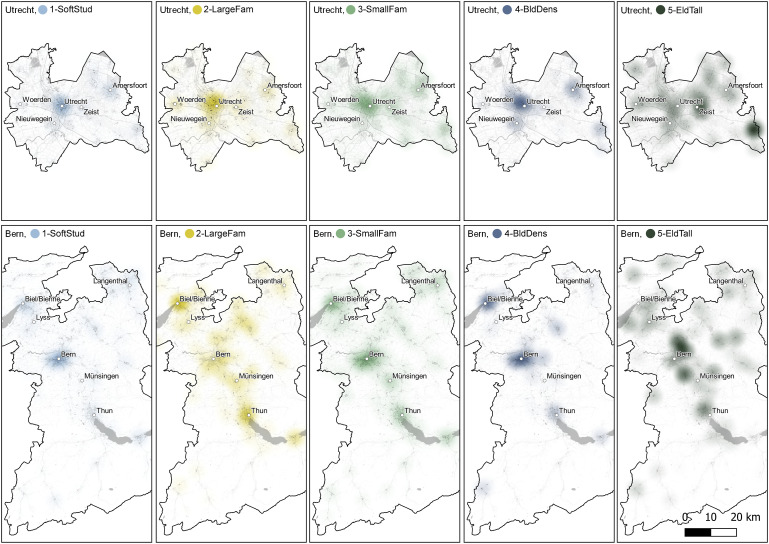
Spatial distribution of densification types in Utrecht and Bern.

Densification is most concentrated in the largest cities, in both case studies (see Figure 4). In the municipalities of Utrecht and Amersfoort, the concentration of new units per hectare is 4 times higher than the average in the province, while in the municipalities of Bern, Thun and Biel/Bienne, the concentration is 14 times higher than the cantonal average (see Table S2). Among all densification types, the types *1-SoftStud* and *4-BldDens* show the highest contrast between the largest cities and the regional average – their concentration in the largest cities is between 5 and 24 times higher than the average (see Table S2). *3-IndivFam* is concentrated predominantly in regional centres, but also in smaller towns. *2-ContFam* developments occur a lot in the centre of Utrecht (4887 units in the municipality). In the centre of Bern, however, they are rather uncommon (125 units in the municipality) and emerged instead outside Bern city centre, along the traffic corridor between Bern and Thun. Still, they did occur inside the smaller towns of Thun and Biel/Bienne. In both case studies, type *5-EldTall* typically occurs in smaller towns and surrounding regional centres. Among all housing units, the units of this type emerged furthest away from the centres of the largest cities (measured as distance from their main stations, see Table S3). There is a remarkable concentration of this type *5-EldTall* in the south-eastern corner of the Province of Utrecht, caused by a reconstruction of two retirement complexes.

## Discussion

We identified five densification types with varying spatial distributions within the case regions between 2011 and 2019. The most striking difference between Utrecht and Bern was the scale of densification projects (distribution of type *2-LargeFam* and *3-SmallFam*). In the following paragraphs, we discuss these results and develop working hypotheses on the possible effects of housing demands and institutional frameworks on these patterns.

### Densification types and their spatial distribution

To a large degree, the identified types and their spatial distribution follow general trends revealed in earlier studies. Densification leads to higher population densities, smaller household sizes, and smaller apartments compared to their urban context, supporting earlier findings by Burton (2000) and Holman et al. (2015). It is most concentrated in the largest cities, whose varied supply of amenities exerts a great attraction to new residents, causing increases in land prices and thus stimulating the transformation of urban land from other purposes to residential use (Broitman and Koomen, 2020; Claassens et al., 2020; Jehling et al., 2020). In addition, the concentration of densification with increased building heights and ground coverage is much higher in the largest cities than the regional average, further steepening urban-rural density gradients (Broitman and Koomen, 2020).

Densification rarely takes place through the transformation of brownfields and urban green spaces, possibly due to complex planning processes or lacking availability (Nabielek, 2011; Nebel et al., 2017). Instead, it is most often an intervention in existing residential areas, both through hard and soft densification, supporting earlier studies in England (Bibby et al., 2020). We observe large-scale densification projects (all the way into the city centre of Utrecht) that could indicate the demolishing and rebuilding of housing blocks, often connected to the displacement of lower-income residents (Rérat et al., 2010; Debrunner and Hartmann, 2020). Additional studies will need to include data on income or rent to complete the picture of potential gentrification effects. On the other end of the spectrum, small-scale, soft densification is highly concentrated in city centres, where it is paired with larger shares of young adults. The growing demand for centrally located apartments among this population group (Rérat, 2019) makes it attractive for private landlords to split and rent apartments to students (Rugg et al., 2002).

However, contradicting the general picture of smaller apartments and household sizes, our analysis found many new housing units that were larger than their surroundings, housing larger households than typical for their neighbourhood. In Bern, comparatively large units are concentrated in smaller towns along the main transport line between Bern and Thun. These developments risk attracting larger households from more to less accessible areas, countering goals for compact urban development (Jehling et al., 2018).

What also could be argued to run counter to densification goals is the comparatively larger living space per person in newly constructed housing units, reducing the added population density compared to what could potentially have been achieved. This contradicts previous concerns that densification diminishes the living space available per person (Burton, 2000; Bibby et al., 2021). Yet, it is well in line with the generally observed rise in per capita living space and simultaneous shrinkage of household sizes – a demand that is catered to in housing construction (Haase et al., 2013).

More specifically, the densification type that shows the highest relative per capita living space is concentrated outside the larger cities and is associated with comparatively tall buildings and high shares of small, elderly households. Perhaps, these seemingly contradicting effects can be explained by the housing demand of an ageing population in less urbanised areas. There, municipalities seek to provide elderly (who occupy large single-family homes) with serviced apartments to make room for younger families (Götze and Hartmann, 2021). More generally, this finding corresponds to the observed housing preferences of elderly residents valuing community attachment and calm environments (Lauf et al., 2012).

Our findings bind together earlier research on resident composition, building characteristics, and transformation processes in the context of densification. In addition to that research, the identified typology reveals processes running in parallel, such as soft densification and large-scale redevelopments. Furthermore, we demonstrate how processes deviate between centre and periphery – different housing types are created to meet different demands. This calls for a more nuanced discussion on the effects of densification (Bibby et al., 2020; Jehling et al., 2020).

### Comparison between Utrecht and Bern

Our results suggest many similarities between Utrecht and Bern regarding the prevalence of densification types and their spatial distribution. The most significant dissimilarity between them concerns type *2-LargeFam*: In Utrecht, 40% of all new housing units were part of large-scale densification projects, compared to only 10% in Bern. In Utrecht, they also occurred in more central locations than in Bern.

On the Dutch side, this can be partially explained by subsidies, granted by the Dutch government in 2007 to redevelop deprived neighbourhoods (Classens et al., 2020). Four of these neighbourhoods are located centrally in Utrecht and overlap with some of the large-scale redevelopments. More generally, in line with the theoretical reasoning on land policies in the section on institutions and urban development, large-scale developments could be enabled by the larger share of corporation owned apartments and the ability of Dutch municipalities to reassemble land through (the threat of) pre-emption and expropriation (Buitelaar and Bregman, 2016). Another reinforcing factor is the cultural expectation that landowners, if unwilling to develop, either sell their land, accept land exchanges, or be persuaded by more attractive development terms (Needham, 2014, p. 13).

In Switzerland, where smaller landowners, rather than corporations, are predominant, large-scale projects are not easily realised. In addition, citizens could be a crucial factor in preventing larger transformations on urban green spaces since re-zoning proposals must legally be put to a vote.

In light of the present arguments, we formulate the following working hypotheses: (1) Active land policy promotes large-scale densification projects, (2) if land is distributed among many owners, individual plots are necessarily smaller on average, which complicates large-scale densification projects (especially given strong property rights), and (3) direct citizen involvement restrains unpopular land use changes (e.g. from urban green space to housing). Further research should be undertaken to investigate these potential institutional factors influencing the scale of densification projects. When discussing densification effects, the project scale is of importance since it can have implications for the loss of green spaces and jobs in industrial sectors, as well as the loss of existing residential building blocks. On the other hand, contiguous redevelopments could also offer opportunities for more holistic planning*.*

### Reflections on the approach

While the approach resulted in convincing comparative findings, some limitations need to be considered for interpretation. For the two cases, national data had to be harmonised to allow for comparability of metrics for social and built densification (section 3.1.1). For social metrics, the hectare-level proved to be adequate in terms of availability and accessibility.

Therefore, we assigned hectare values to each housing unit. These values represent well the residents of newly constructed housing units in case many units were constructed in a hectare cell. However, if only a few units were constructed, the hectare value is strongly influenced by pre-existing social structures. Since each housing unit represents one data point, we argue that large densification projects of many housing units are more weighted in the analysis, thus compensating for this error.

A similar error occurred due to the mutual aggregation of land use to hectare cells. For instance, this led to wrongly attributing housing units in residential areas next to parks as ‘transformation of urban green spaces’. Since the transformation type is an important variable in the characterisation of densification types, we covered for that through only considering units at locations where land use has changed to ‘residential’ or ‘construction site’ by 2019. However, the latest land use datasets are from 2018 (Switzerland) and 2015 (the Netherlands), respectively. Therefore, housing units on brownfields or urban green spaces constructed towards the end of the period are filtered out as well. In total, around 30% of all new housing units (including expansion) in Utrecht and 40% of all new housing units in Bern were filtered out (see Figure S5).

Another concern regards the comparability of urban development in two regions with substantially different degrees of urbanisation, age distributions, building traditions and landscapes. As a possible solution to this issue, we proposed metrics that characterise densification types in relation to their direct surroundings (Jehling and Hecht, 2021). We expressed densification as deviations from average values in a circle of 1 km^2^ around each new housing unit. The results provide relevant information on the changes that densification inflicts on a neighbourhood, independent of whether absolute values are the same in two regions. However, by using this approach, we pay the price of not being able to make any statements about absolute values. Still, since there is a strong correlation between absolute and relative values in our data, we can assume that an apartment that is larger than its surroundings is also a fairly large apartment.

Finally, the clustering algorithm chosen in this study had the advantage that the categorical variable ‘transformation process’ could be included in the typology. K-proto cluster analyses are not being used very frequently, although, to a large part, they resemble the better known k-means method (Bobkova et al., 2021). Since, in our analysis, the simple distance measure was weighted by a relatively small factor γ = 1.4, it results in mean values for clusters similar to k-means analysis, as it has been tested on the continuous variables of the same data set. In addition, visualising the continuous variables of this study using principal components shows a reasonable delineation between the five densification types (see Object S6 for ordination with three principal components). Therefore, we assess that k-proto is a promising method to cluster large mixed-type datasets for urban analysis.

## Conclusion

We presented an approach to characterise and compare social and building changes from urban densification, providing an empirical basis for policy evaluation. The method makes use of national datasets on socio-demography, residential buildings and land use changes. A k-proto cluster analysis revealed densification types describing (1) how new housing units deviate from their urban context, (2) how residents in new housing units deviate from their urban context, and (3) the construction process regarding the average project size and the transformation process through which the new housing units emerged. By describing densification in relation to its urban context, this method enables international comparison. We successfully applied it to the cases of Utrecht (the Netherlands) and Bern (Switzerland) between 2011 and 2019 and identified five densification types whose frequency substantially deviates between the cases. Most strikingly, large-scale densification projects occur more often in Utrecht than in Bern, likely due to the prevalence of larger actors in the real-estate market in combination with the ability of Dutch municipalities to apply strong instruments of active land policy. Future research could usefully explore the role of real-estate market players in shaping densification patterns. Our findings suggest that the approach presented here is suitable for addressing causal relations between institutions (especially property rights and land policies) and densification. Furthermore, the approach revealed a diverse array of densification types and outcomes, challenging the widely held assumption that densification is a monolithic phenomenon. Ergo, a more differentiated discussion of the social, economic and ecological effects of densification is necessary.

## Supplemental Material

Supplemental Material - Comparing types and patterns: A context-oriented approach to densification in Switzerland and the Netherlands Supplemental Material for Comparing types and patterns: A context-oriented approach to densification in Switzerland and the Netherlands by Vera Gotze, Mathias Jehling in Environment and Planning B: Urban Analytics and City Science
