# Supplementary material for: Comparing types and patterns: A context-oriented approach to densification in Switzerland and the Netherlands
Source: Environ Plan B Urban Anal City Sci. 2022 Dec 2;50(6):1645–59. doi: 10.1177/23998083221142198 (PMC13032841; doi:10.1177/23998083221142198)
Supplement: Supplemental Material - Comparing types and patterns: A context-oriented approach to densification in Switzerland and the Netherlands [file sj-pdf-1-epb-10.1177_23998083221142198.pdf]

Supplementary material to *„Comparing types and patterns: A context-oriented approach to densification in Switzerland and the Netherlands"*

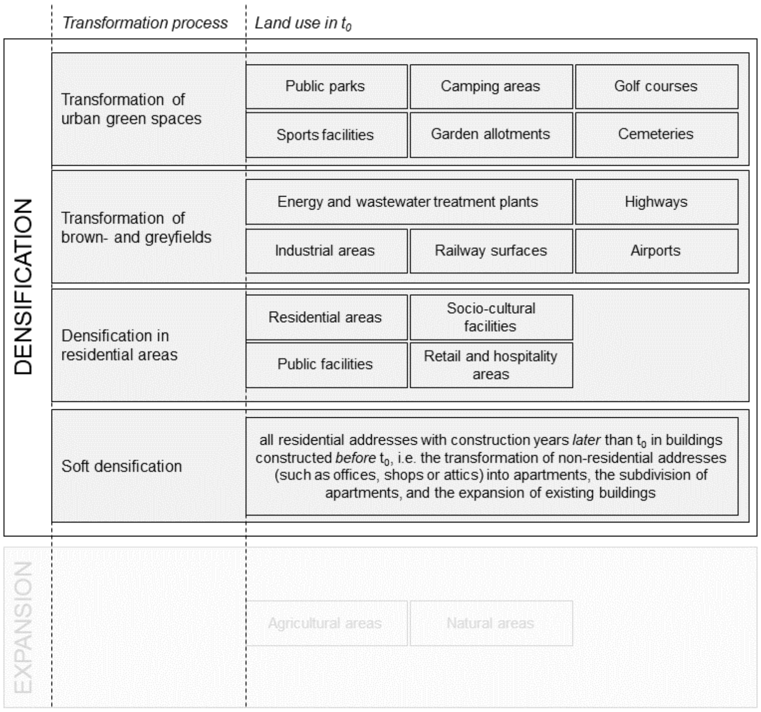

Figure S1: Transformation processes by former land use (at t_0).

Table S1: Metrics for densification with minimum, mean and maximum values.

	Name	Description	Aggregation level	Min	Mean	Max
SOCIAL	pop_dens	Inhabitants per hectare	Hectare grid	3	91.6	484
	per_kids	Share of children (age 14 and below) per hectare	Hectare grid	0	14.2	100
	per_students	Share of students (age 15 to 24) per hectare	Hectare grid	0	9.7	100
	per_elderly	Share of elderly (age 65 and above) per hectare	Hectare grid	0	20.9	100
	hh_size	Average household size per hectare	Hectare grid	1	2.1	6
	m2person	Sum of apartment sizes divided by population per hectare	Hectare grid	11.8	61.3	494.5
BUILT	layers	Average number of storeys per street block	Street block	1	2.4	9
	fsi	Ratio between floor area and block area per street block	Street block	0.01	0.7	5.7
	gsi	Ratio between building footprint and block area per street block	Street block	0.01	0.3	0.99
	aptsize	Apartment sizes of individual apartments	Housing unit	11	98.9	495
SOCIAL	d_popdens	Deviance in population density from surroundings	Hectare grid	-1.9	0.4	7.2
	d_kids	Deviance in share of children from surroundings	Hectare grid	-3.4	0.1	6.7
	d_students	Deviance in share of students from surroundings	Hectare grid	-2.9	-0.03	10.1
	d_elderly	Deviance in share of elderly from surroundings	Hectare grid	-3.8	-0.06	5.7
	d_hh_size	Deviance in household size from surroundings	Hectare grid	-4.4	-0.2	6.3
	d_m2person	Deviance in living space per person from surroundings	Hectare grid	-5.9	0.2	12.4
BUILT	d_layers	Deviance in building height from surroundings	Street block	-3.1	0.3	7.2
	d_fsi	Deviance in floor space index from surroundings	Street block	-2.7	0.3	7
	d_gsi	Deviance in ground space index from surroundings	Street block	-2.6	0.2	4.6
	d_aptsize	Deviance in apartment size from surroundings	Housing unit	-4	-0.01	7.3
PROCESS	patchsize	Size of densification project in m^2	Housing unit	87.7	5857	90381
	transformation type	Factor with levels: transformation of brownfields/greyfields, transformation of urban green, densification on residential areas, soft densification	Hectare grid	-	-	-

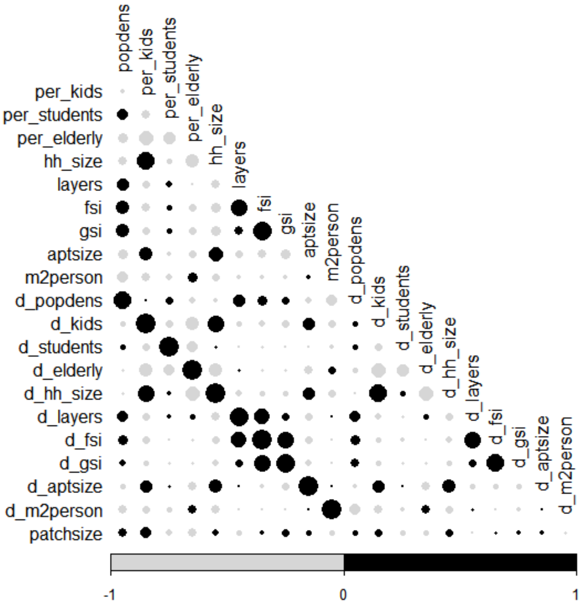

Figure S2: Correlogram of variables describing densification in Utrecht and Bern. The point size represents the strength of the correlation (larger = stronger).

Correlation analysis is conducted to validate variable selection and reduce variables for cluster analysis. The correlation analysis (see Figure S2) sustains the decision to use urban context-oriented variables (see deviation variables in Table 1), as strong correlations exist between relative and total values. Within this set of urban context-oriented variables, strong positive correlations additionally justify the reduction of morphological metrics to *d_layers* and *d_gsi* as they are relevant for discussing sensitive topics, such as high-rises and the amount of free space. Another significant, positive correlation occurs between average household sizes and the share of children in hectare grid cells (*d_hh_size* and *d_kids* with r = 0.7). As the age range 0 to 14 does not cover all children living at home, we keep household sizes as a more precise indicator for the share of families with children. Finally, the size of a densification project (*patchsize*) shows no significant correlation with other variables and is kept as an important measure for the scale of densification.

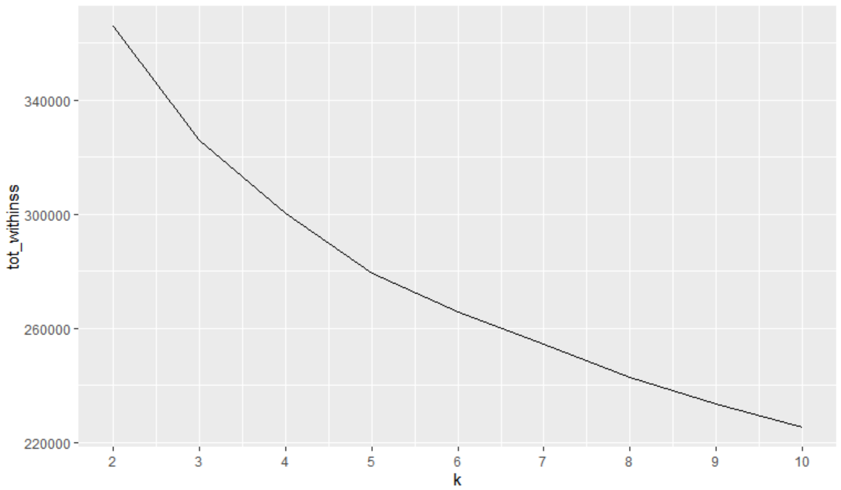

Figure S3: Scree plot showing the decrease in total within sum of squares distance per k.

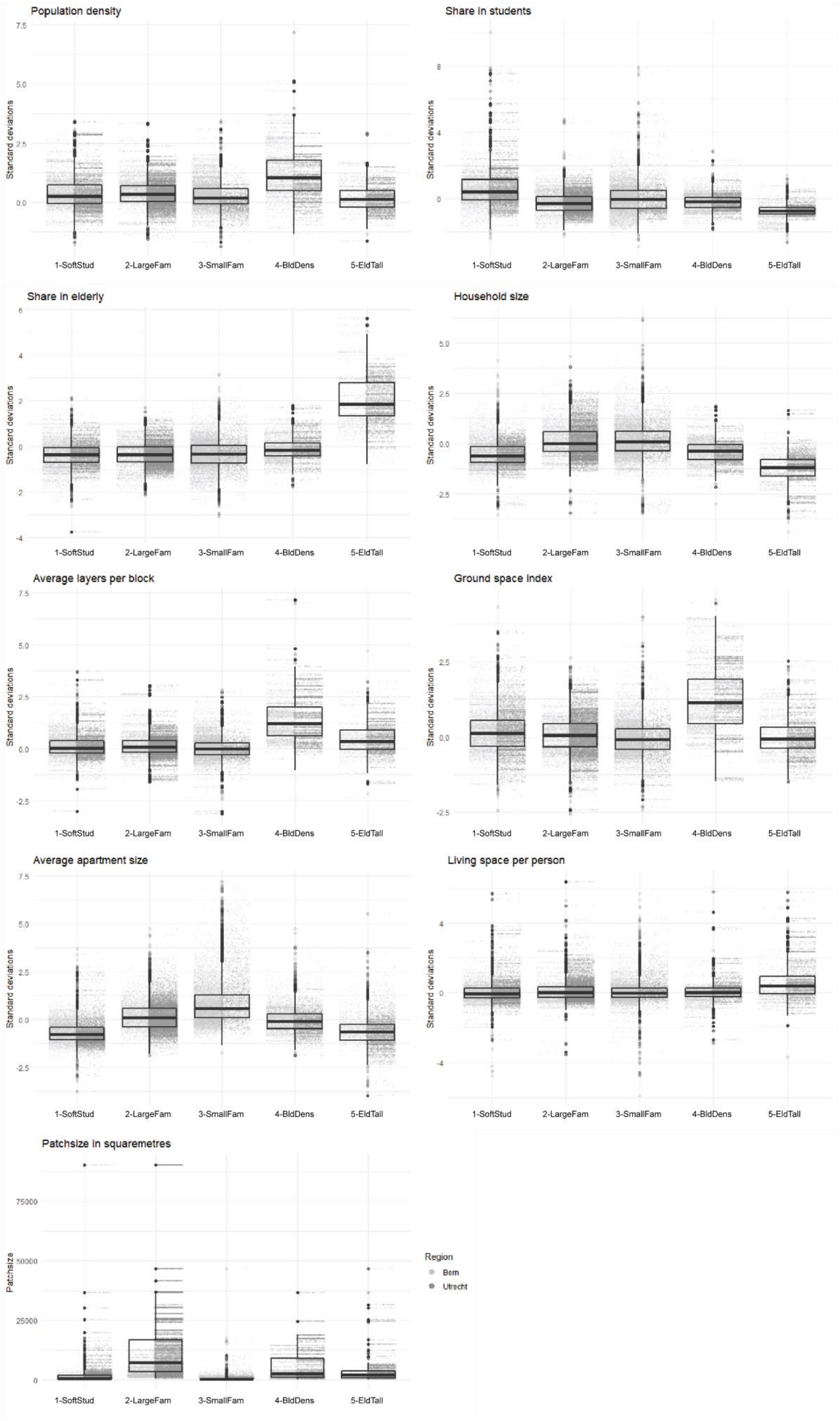

Figure S4: Distribution of observations per metric and densification type for Bern and Utrecht

Table S2: Concentration of densification types within and outside of largest municipalities

**Canton of Bern** (Area: 582.826 ha)					
	Total in Canton of Bern	*Units per ha in Canton*	*Total in municipalities of Thun, Biel, Bern*	*Units per ha in Thun, Biel, Bern (9.436 ha)*	*Urban to non-urban ratio*
1-SoftStud	4778	0.008	1040	0.110	13.4
2-LargeFam	4363	0.007	619	0.066	8.8
3-SmallFam	9500	0.016	1693	0.179	11.0
4-BldDens	3881	0.007	1789	0.190	28.5
5-EldTall	1383	0.002	77	0.008	3.4
Total	23006	0.039	5213	0.552	14.0
**Province of Utrecht** (Area: 150.000 ha)					
	Total in Province of Utrecht	*Units per ha in Province*	*Total in municipalities of Utrecht and Amersfoort*	*Units per ha in Utrecht, Amersfoort (16.307 ha)*	*Urban to non-urban ratio*
1-SoftStud	7435	0.050	3809	0.234	4.7
2-LargeFam	13908	0.093	5825	0.357	3.9
3-SmallFam	2506	0.017	905	0.055	3.3
4-BldDens	4183	0.028	2317	0.142	5.1
5-EldTall	4591	0.031	745	0.046	1.5
Total	32623	0.217	13601	0.834	3.8

Table S3: Median distance of densification types to central stations

	Median distance to nearest of the main stations of Biel, Thun or Bern	*Median distance to nearest of the main stations of Utrecht or Amersfoort*
1-SoftStud	10.4	4.4
2-LargeFam	8.8	7.3
3-SmallFam	10.1	7.8
4-BldDens	3.4	4.4
5-EldTall	10	9.6
Total	8.6	7

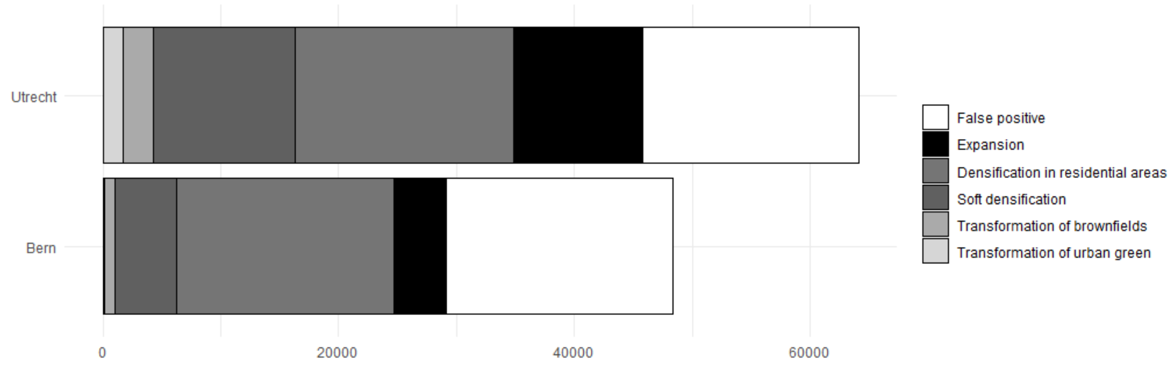

Figure S5: Total new housing units between 2011 and 2019 by transformation process, including false positives.
